# The Diagnostic Value of ^18^F-FDG PET/CT Bone Marrow Uptake Pattern in Detecting Bone Marrow Involvement in Pediatric Neuroblastoma Patients

**DOI:** 10.1155/2022/7556315

**Published:** 2022-01-06

**Authors:** Jun Liu, Cuicui Li, Xu Yang, Xia Lu, Mingyu Zhang, Luodan Qian, Wei Wang, Ying Kan, Jigang Yang

**Affiliations:** Department of Nuclear Medicine, Beijing Friendship Hospital, Capital Medical University, Beijing 100050, China

## Abstract

**Objectives:**

To explore the diagnostic value of ^18^F-FDG PET/CT bone marrow uptake pattern (BMUP) in detecting bone marrow involvement (BMI) in pediatric neuroblastoma (NB) patients.

**Methods:**

Ninety-eight NB patients were enrolled in BMI analysis. Four patterns of bone marrow uptake were categorized based on pretreatment ^c^F-FDG PET/CT images. Some crucial inspection indexes and ^18^F-FDG PET/CT metabolic parameters were analyzed. The BMUP was divided into BMUP1, BMUP2, BMUP3, and BMUP4. Paired-like homeobox 2b (PHOX2B) of bone marrow and blood, bone marrow biopsy (BMB) result, and ^18^F-FDG PET/CT were compared to detect BMI. All patients were followed up for at least six months.

**Results:**

BMUP had excellent consistency among different physicians. Kappa coefficients of two residents and two attending physicians and between the resident and attending physician, were 0.857, 0.891, and 0.845, respectively. The optimal cut-off value of SUVmax-Bone/Liver was 2.08 to diagnose BMI for BMUP3 patients, and the area under curve (AUC) was 0.873. AUC of PHOX2B of bone marrow (PHOX2B of BM), PHOX2B of blood, BMB, and ^18^F-FDG PET/CT were 0.916, 0.811, 0.806, and 0.904, respectively. There was no significant difference between PHOX2B of BM and PET/CT. Positive predictive value, negative predictive value, sensitivity, and specificity in diagnosis of BMI were 92.9%, 92.9%, 97.0%, and 83.9% for PET/CT and 96.7%, 80.6%, 89.6%, and 93.5% for PHOX2B of BM, respectively.

**Conclusions:**

BMUP of pretreatment ^18^F-FDG PET/CT is a simple and practical method, which has a relatively high diagnostic efficiency in detecting BMI and might decrease unnecessary invasive inspections in some pediatric NB patients.

## 1. Introduction

Neuroblastoma (NB) is one of the most common malignant tumors in children, which originates from neural-crest cells, and related with about 12% to 15% of pediatric cancer deaths [1, 2]. Approximately 50% of NB patients show extensive metastases at initial diagnosis, which would have a poor prognosis [3, 4]. Bone marrow is the most common metastasis site of NB, and patients without bone marrow involvement (BMI) would have a more favorable prognosis than those with BMI [5]. Moreover, accurate assessment of BMI is essential for correcting stage and risk stratification [6].

Cytology of aspirates and histology of biopsies have been the gold standard to evaluate BMI in NB for many years. However, these methods have limited sensitivity when NB involvement is less than 10% and could seriously underestimate the prevalence of BMI [5, 7, 8]. As an invasive method, bone marrow biopsy (BMB) only contains a limited part of bone marrow, which might miss the region of malignant infiltration and should not be considered as a gold standard in childhood malignancy [9, 10]. More precise and sensitive methods for detecting BMI have been developed in the past few decades. Currently, there are some techniques to precisely detect BMI, including immunocytology, multiparameter flow cytometry, and reverse transcriptase-quantitative polymerase chain reaction (RT-qPCR). Paired-like homeobox 2b (PHOX2B) is one of the most sensitive and specific tumor-related biomarkers, which could be detected by RT-qPCR to evaluate minimal metastatic or residual lesions of NB [5, 11, 12].

^18^F-FDG PET/CT, as a major functional imaging method, has been widely used in pediatric tumors for diagnosis and staging [6, 13, 14]. The recently revised International Neuroblastoma Response Criteria (INRC) has emphasized the role of ^18^F-FDG PET/CT in evaluating NB stage and predicting prognosis, especially for patients who are non-MIBG avid [2]. ^18^F-FDG PET/CT has been a sensitive method to detect BMI for many malignancies. Most of those researches are based on lymphoma patients' data [7, 15, 16]. Few researchers have evaluated the diagnostic value of ^18^F-FDG PET/CT in diagnosing BMI in NB.

The aim of the present study was to investigate the value of pretreatment ^18^F-FDG PET/CT bone marrow uptake pattern (BMUP) in detecting BMI in newly diagnosed pediatric NB and compared it with bone marrow biopsy and PHOX2B.

## 2. Materials and Methods

### 2.1. Patients

**Figure 1:**
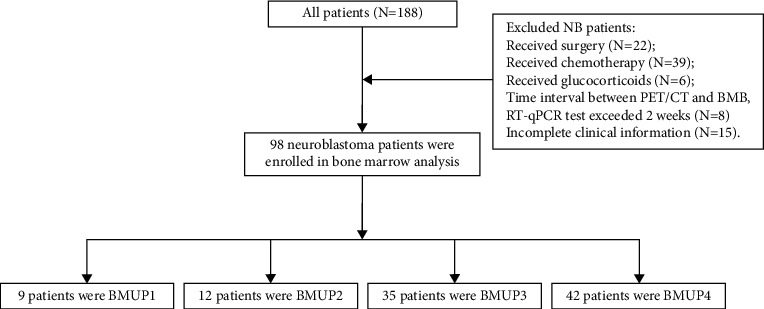
Flow diagrams of study populations.

We retrospectively analyzed the medical record of pediatric patients (age <18 years old) from January 2018 to December 2019 in the nuclear medicine department, Beijing Friendship Hospital, Capital Medical University. A total of 188 NB patients were identified from the electronic patient files. This study followed the inclusion criteria: (1) newly diagnosed pediatric NB patients performed ^18^F-FDG PET/CT scan before surgery; (2) ^18^F-FDG PET/CT, bone marrow testing, and RT-qPCR test were performed within two weeks. The main exclusion criteria included the following: (1) NB patients receiving chemotherapy, glucocorticoids, or hematopoietic cytokines before ^18^F-FDG PET/CT; (2) patients with severe active inflammation (Figure 1). The NB stage was based on the International Neuroblastoma Risk Group Staging System (INRGSS).

Clinical data of NB patients were obtained from medical records, including age, gender, weight, height, primary tumor site, and follow-up information. All patients must be followed up for six months or more. The permission to conduct this retrospective study was obtained from the Institutional Review Board of Beijing Friendship Hospital, Capital Medical University, which waived the requirement of informed consent (L-2019-18).

### 2.2. ^18^F-FDG PET/CT Protocol

All ^18^F-FDG PET/CT imaging were acquired from Siemens Biograph 64-slice mCT (Siemens Medical Systems, Germany) according to the guidelines recommended by the European Association of Nuclear Medicine in pediatric oncology [17]. Patients were fasted at least six hours before ^18^F-FDG PET/CT scan, and ^18^F-FDG would be injected when blood glucose level was <200 mg/dL. Patients were scanned approximately one hour after injection of ^18^F-FDG at the dose of 3.7 MBq/kg, and all ^18^F-FDG PET/CT scans were performed with low dose CT, ranging from the head to the mid-thighs or feet without contrast medium. Sedation would be carried out approximately 30 mins before scanning if pediatric patients were unable to cooperate.

### 2.3. ^18^F-FDG PET/CT Image Review

All images were independently reviewed by two groups of nuclear medicine physicians (two residents and two attending physicians), blinded to laboratory indexes, clinical information, and BMB results. Disagreements were discussed and adjudicated through a third physician.

**Figure 2:**
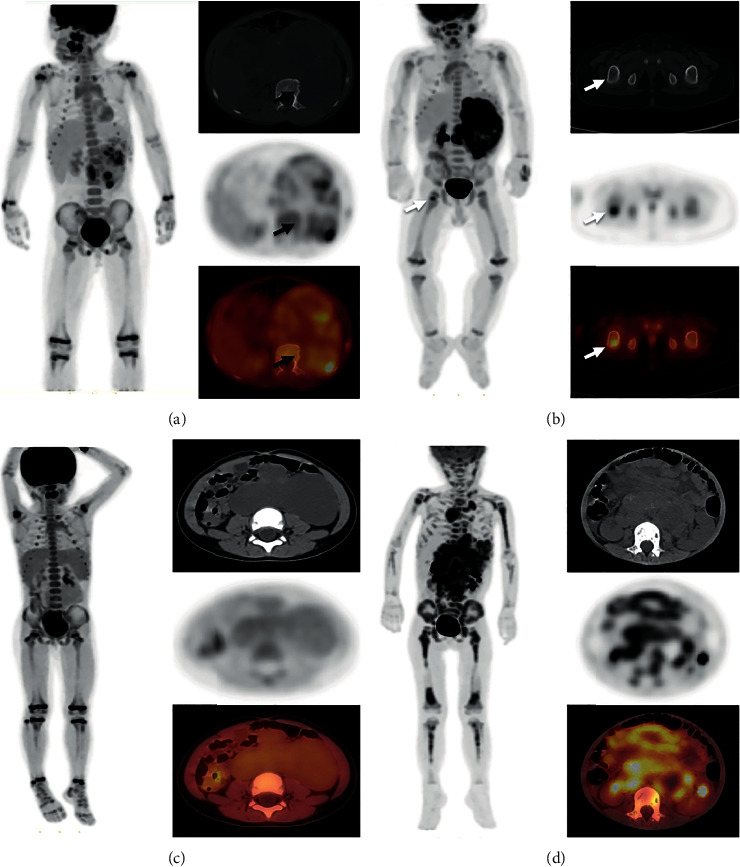
^18^F-FDG PET/CT bone marrow uptake pattern (BMUP). (a) BMUP1 was defined as direct invasion or being surrounded by adjacent tumor tissue (black arrow). (b) BMUP2 was defined as focal/multifocal uptake (white arrow), which was one or more (less than 30) restricted area(s) of FDG uptake. (c) BMUP3 was defined as diffuse and homogeneously increased FDG uptake within the skeleton. (d) BMUP4 was defined as diffuse and heterogeneously increased FDG uptake within the skeleton.

Based on our routine clinical practice experience, the general form of bone/bone marrow metastasis, and previous researches [8], all patients were classified into four BMUP according to the bone marrow uptake pattern of ^18^F-FDG PET/CT before treatment. BMUP1 was defined as direct invasion or being surrounded by adjacent tumor tissue (Figure 2(a)). BMUP2 was defined as focal/multifocal uptake, which was located in one or more restricted area(s) of FDG uptake (Figure 2(b)); BMUP3 was defined as diffuse and homogeneous FDG uptake in the skeleton (Figure 2(c)); BMUP4 was defined as diffused and heterogeneous FDG uptake in the skeleton (Figure 2(d)).

### 2.4. Identification of BMI

Bone marrow aspiration and biopsy at unilateral iliac crest were performed routinely at the time of initial diagnosis of NB. BMB would be tested using normal cytology, which was based on standard immunohistochemistry or morphological analysis of bone marrow aspirates. PHOX2B of bone marrow (PHOX2B of BM) and peripheral blood would be tested through RT-qPCR, which were expressed as the fold change in PHOX2B gene expression relative to the healthy controls. All patients were classified into four BMUP, according to the ^18^F-FDG PET/CT uptake patterns of bone marrow. Since children were unlikely to develop degenerative diseases, those typical focal and heterogeneous FDG uptake patterns were easy to diagnose BMI in pediatric patients, so patients with BMUP1, BMUP2, and BMUP4 were interpreted as having BMI on ^18^F-FDG PET/CT images. BMI was hard to identify in patients with homogeneous FDG uptake, so further evaluation of BMUP3 was required. If only one of the four test methods were positive, we would review the positive result and check again.

### 2.5. Semiquantitative ^18^F-FDG PET/CT Parameters for BMUP3

The diffuse and homogeneous ^18^F-FDG uptake may be caused by tumor infiltration or myeloid hyperplasia reaction. In order to differentiate reactive uptake in BMUP3, semiquantitative ^18^F-FDG PET/CT parameters would be measured. A circular region of interest was placed in the normal area of left and right lobe of liver, respectively. The average SUVmax value of two regions was considered as the maximum standardized uptake value of liver (SUVmax-Liver). A spherical volume of interest was placed in L1 to L5 vertebral body, and the maximum standardized uptake value of lumbar spine was determined as SUVmax-Bone. Then, the ratio of SUVmax-Bone to SUVmax-Liver (SUVmax-Bone/Liver) was calculated.

### 2.6. Final Diagnosis

The presence of BMI was based on one or more results of the following: (1) directed biopsies guided by PET/CT were confirmed positive; (2) supplementary targeted other diagnostic imaging (including enhanced CT, MRI, and ^123^I-MIBG SPECT/CT) within two weeks confirmed positive for corresponding site; (3) regression of bone marrow lesions in parallel with other neuroblastoma lesions on clinical follow-up (including the imaging results and relevant laboratory tests).

### 2.7. Statistical Analysis

Continuous data were expressed as mean ± standard deviation (mean ± SD), and categorical data were expressed as numbers (percentages). Clinical characteristic factors and diagnostic accuracy of different BMUP groups were compared by using Student's *t*-tests, Mann–Whitney *U* test, Pearson's Chi-square test, and Fisher's exact test as appropriate. Multiple comparisons between different BMUP groups were corrected using the Bonferroni method, and the interobserver agreement was evaluated by Cohen's Kappa statistic. Optimal cut-off values were analyzed using receiver operating characteristic (ROC) curves and Youden index in BMUP3 NB patients for diagnosing BMI. The diagnostic efficacy of the three tests and ^18^F-FDG PET/CT were evaluated by comparing areas under ROC curves. Statistical analyses were performed using SPSS 22.0 and MedCalc 12.7.0. The two-sided test was selected, and a *P* value＜0.05 was considered statistically significant.

## 3. Results

### 3.1. BMUP Diagnostic Consistency

Ninety-eight pediatric patients (48 boys and 50 girls) with newly diagnosed NB were finally enrolled in our study. In order to evaluate different observer agreements for BMUP, we divided the observers into two groups (two residents and two attending physicians). Kappa coefficients of two residents and two attending physicians were 0.857 (95%CI: 0.793–0.957) and 0.891 (95%CI: 0.815–0.968), respectively. The kappa coefficient between resident group and attending physician group was 0.845 (95%CI: 0.756–0.935). All groups had excellent kappa coefficients, indicating that BMUP was a simple and easy method for diagnosis BMI in pediatric NB. Results are summarized in Table 1.

### 3.2. Clinical Characteristics

According to the classification of attending physician group, there were 9, 12, 35, and 42 patients in BMUP1, BMUP2, BMUP3, and BMUP4, respectively. The mean age of patients was 3.53 ± 2.17 years old, and about 79.6% (78/98) of pediatric patients were younger than five years old. Most patients (68/98, 69.4%) had advanced stage disease (stage M), and 11 patients (11/98, 11.2%) were early-stage (stage L1). The tumors of 83.6% (82/98) of the patients were located in the abdomen, while only three patients had the tumor in the pelvic cavity. In this study, despite significant differences in age, primary sites, INRGSS, and International Neuroblastoma Risk Group classification system (INRGCS) among different BMUP, the patient's gender did not show significant difference to the pattern of uptake. Clinical data of these patients are shown in Table 2 and Supplementary Table 1.

### 3.3. Semiquantitative Parameter Evaluation for BMUP3

**Figure 3:**
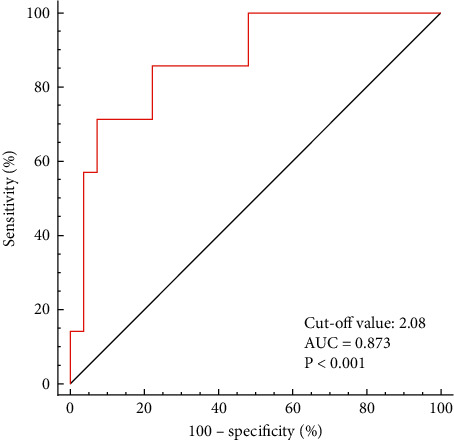
Receiver operating characteristic curve of SUVmax-Bone/Liver for BMUP3 patients.

Semiquantitative parameters of ^18^F-FDG PET/CT were used to assess BMI in BMUP3 patients. The optimal cut-off value of SUVmax-Bone/Liver for distinguishing metastasis from reactive uptake in BMUP3 patients was 2.08, and the area under curve (AUC) was 0.873 (95% CI: 0.714–0.962, *P* < 0.001). With 2.08 as the cut-off value, seven patients had BMI, and 28 patients had no bone marrow metastases (one child having diffuse liver metastases could not have its SUVmax-Bone/Liver calculated). The sensitivity, specificity, and accuracy of cut-off values were 71.4%, 92.6%, and 88.6%, respectively. When we adjusted the cut-off value to 1.5 or 3.0, the sensitivity and specificity of the cut-off values were 100.0% and 33.3% or 14.3% and 100.0%, respectively. The results are shown in Figure 3, Supplementary Table 2, and Supplementary Table 3.

The optimal cut-off value of SUVmax-Bone/Liver was 2.08, and areas under curve (AUC) was 0.873 (95% CI: 0.714–0.962, *P* < 0.001).

### 3.4. Evaluation of BMI

**Figure 4:**
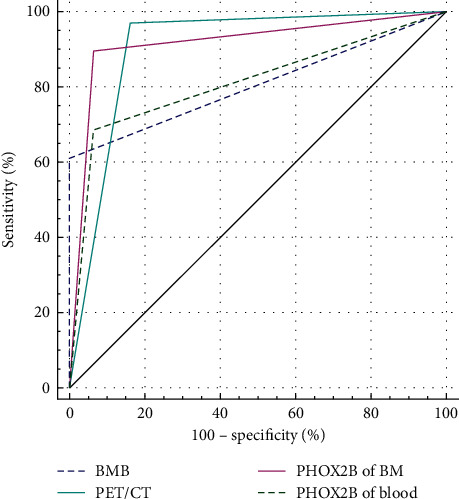
Diagnostic efficiency between ^18^F-FDG PET/CT with other three tests in all pediatric NB patients.

There were 70 positive patients and 28 negative patients in ^18^F-FDG PET/CT, 41 positive patients and 57 negative patients in BMB, 48 positive patients and 50 negative patients in PHOX2B of blood, and 62 positive patients and 36 negative patients in PHOX2B of BM. Positive predictive value (PPV), negative predictive value (NPV), sensitivity, and specificity, were 92.9%, 92.9%, 97.0%, and 83.9% for ^18^F-FDG PET/CT and 96.7%, 80.6%, 89.6%, and 93.5% for PHOX2B of BM, respectively (Table 3). We compared the diagnostic efficiency between ^18^F-FDG PET/CT with another three tests in NB patients. The AUC of PHOX2B of BM was 0.916, which was highest compared with the other two tests and ^18^F-FDG PET/CT, while it did not show a significant difference with ^18^F-FDG PET/CT (AUC = 0.904, *p* = 0.817). ^18^F-FDG PET/CT was better than BMB (AUC = 0.806, *p* = 0.028) with a significant difference and better than PHOX2B of blood (AUC = 0.811, *p* = 0.078) with a marginal significant difference. Details are shown in Table 4 and Figure 4.

Areas under curve (AUC) were 0.904 (95% CI: 0.828–0.955) for ^18^F-FDG PET/CT, 0.916 (95% CI: 0.842–0.962) for PHOX2B of BM, 0.811 (95% CI: 0.719–0.883) for PHOX2B of blood, and 0.806 (95% CI: 0.714–0.879) for BMB.

We further investigated the diagnostic accuracy of ^18^F-FDG PET/CT, BMB, PHOX2B of blood, and PHOX2B of BM in the different BMUP. According to the BUMP grouping, the diagnostic accuracy of BMI in BMUP1, BMUP2, BMUP3, and BMUP4 of FDG PET were 66.7% (6/9), 100.0% (12/12), 88.6% (31/35), and 100.0% (42/42), in BMB were 33.3% (3/9), 16.7% (2/12), 80.0% (28/35), and 92.8% (39/42), in PHOX2B of blood were 33.3% (3/9), 33.3% (4/12), 82.9% (29/35), and 92.8% (39/42), and in PHOX2B of BM were 77.8% (7/9), 83.3% (10/12), 85.7% (30/35), and 100.0% (42/42). ^18^F-FDG PET/CT was better than BMB and PHOX2B of blood in diagnosing BMI in BMUP2 patients with significant difference, and the results are shown in Table 5.

## 4. Discussion

We proposed ^18^F-FDG PET/CT BMUP and evaluated the value of BMUP in detecting BMI compared with PHOX2B of blood and PHOX2B of BM in newly diagnosed NB. Pretreatment ^18^F-FDG PET/CT BMUP was a simple method for diagnosing BMI in pediatric NB patients, which had excellent coefficients between different physicians. We demonstrated that the optimal cut-off value was 2.08 for SUVmax-Bone/Liver of BMUP3 to diagnose BMI. The PHOX2B of BM had the highest AUC in diagnosing BMI, but there was no significant difference with ^18^F-FDG PET/CT. ^18^F-FDG PET/CT was better than normal BMB and PHOX2B of blood in detecting BMI, especially in BMUP2.

NB is a common neuroectodermal tumor, which originates from the neural crest, while the bone marrow was the most metastatic site of NB [18]. BMI was a sign of widespread tumor involvement and indicated a higher stage, while tumor infiltration in bone marrow could be heterogeneously distributed throughout the skeleton in NB [2]. Some studies have summarized the performance of bone marrow infiltration and proposed bone marrow uptake patterns, while those studies were mainly based on lymphoma, and there were only a few studies on the bone marrow uptake patterns of NB [8, 15, 19, 20]. Li C et al. classified the patterns of bone marrow ^18^F-FDG uptake on PET/CT into three types; these patterns were easily confused and difficult to distinguish in clinical practice [8]. In our research, we combined previous research results and our clinical experience to propose four bone marrow uptake patterns, which were more simple and easier to learn. We have tested the consistency of four bone marrow uptake patterns among different physicians (resident group and attending physician group), which showed an excellent level of interobserver agreement. Our research results showed better acceptability in clinical practice, and ^18^F-FDG PET/CT BMUP would be an easy way to detect BMI.

There was direct evidence of BMI in PET/CT images, for BMUP1, BMUP2, and BMUP4. However, for diffuse and homogeneous FDG uptake (BMUP3), there was still lack of standardized methods to assess BMI in PET/CT images. For children especially, the diffuse and homogeneous uptake of bone marrow may be caused by paraneoplastic bone marrow activation, cancerous infiltration, and inflammatory reaction or as a result of hematopoietic growth factors [8, 21]. Normally, BM showed homogeneous uptake lower than liver, and the uptake of ^18^F-FDG is consistent with the distribution of red bone marrow [15, 21]. Many previous studies often distinguished BMI from normal bone marrow uptake when bone marrow ^18^F-FDG uptake was higher than liver, especially in lymphoma patients [9, 22, 23]. While many pediatric patients' bone marrow uptake was higher than liver, as shown in our study, this may be due to the younger patients having active bone marrow hematopoiesis. Differentiating BMI from normal/reactive uptake was very crucial for further management and therapy. We adopted a semiquantitative approach for further judgment by using the ratio of SUVmax-Bone to SUVmax-Liver. We demonstrated that the optimal cut-off value was 2.08 for SUVmax-Bone/Liver to diagnose BMI in BMUP3, which was higher than liver uptake and different from the previous study [8]. Hopefully, this may reduce the incidence of false positive in younger NB patients that might cause by physiological or pathological reasons.

BMI was an important prognostic factor for NB; however, there was still much controversy about detecting BMI [9, 24]. In the past several decades, BMB positivity was the gold standard for BMI [24]. As a heterogeneous tumor, NB infiltration in the bone marrow could be heterogeneously distributed throughout the skeleton [2]. Whether or not the BM was involved could not be judged only by bone marrow aspiration, because it only analyzed very limited places in the iliac crest and might miss lesions, which were located elsewhere [8]. The results of bone marrow aspirates were also susceptible to the sample quality; hence, quality control of bone marrow biopsy specimens was recommended by newly revised guidelines [2, 5]. For NB patients, the conventional histology and cytology tests would not always be positive, if the level of tumor cells in the bone marrow was less than 1% [25]. In the past several decades, RT-qPCR had been a more sensitive way to detect bone marrow metastatic lesions and was successfully applied in various tumors [26–28]. There were several RT-qPCR molecular biomarkers for NB. Among these molecular biomarkers, the PHOX2B in bone marrow and peripheral blood had the best diagnostic performance and was considered to be the most optimal biomarker for BMI detection [29–31]. Some previous researches had confirmed the value of ^18^F-FDG PET/CT in detecting BMI compared with BMB in lymphoma [32–35]. However, few studies had explored the role of ^18^F-FDG PET/CT in NB [8], and there was no study comparing PHOX2B with ^18^F-FDG PET/CT in NB. Our present study compared ^18^F-FDG PET/CT with PHOX2B and BMB, and PET/CT showed no inferior diagnostic performance to PHOX2B of BM. In BMUP2 patients especially, ^18^F-FDG PET/CT showed better diagnostic efficacy than BMB and PHOX2B of peripheral blood. Detection of BMI by BMB or PHOX2B of peripheral blood might be omitted, or a PET/CT-guided bone marrow biopsy should be performed to decrease the harm caused by unnecessary bone marrow puncture biopsy in BMUP2 patients, partly similar to previous studies [8]. In general, ^18^F-FDG PET/CT BMUP was a more convenient and noninvasive method to diagnose BMI for pediatric NB patients and might decrease unnecessary bone marrow aspiration in some pediatric NB patients.

### 4.1. Limitation

There were some limitations in the present study. First, this was a retrospective single-center study with a small sample. Second, we only took samples from the unilateral iliac crest for RT-qPCR testing. For pediatric NB patients, it was difficult for us to perform multiple bone marrow biopsies as recommended. Third, there was no acceptable way to confirm every lesion showing in PET/CT, especially in our pediatric NB patients. Then, in order to avoid the impact of bone marrow puncture of the ilium on SUVmax-Bone in PET/CT, we adopted the method of drawing ROI on the lumbar spine and abandoned the method of drawing ROI on the iliac bone, which might better reflect bone marrow pathology. Finally, the SUVmax-Bone/Liver >2.08 for predicting BMI in BMUP3 is only obtained from limited data in our single institute, which may lack universal representation, and the final diagnosis of BMI still has some shortcomings, which might need further refinement. A large prospective multi-center PET/CT multiparameter study should be taken to validate our findings in the future.

## 5. Conclusion

^18^F-FDG PET/CT BMUP is a simple and practical method to evaluate BMI and has a relatively high diagnostic efficiency. ^18^F-FDG PET/CT could play a complementary role in accurately diagnosing BMI and decrease unnecessary invasive inspections for some pediatric NB patients.

## Figures and Tables

**Table 1:** ^18^F-FDG PET/CT bone marrow uptake pattern diagnostic consistency in different physicians.

		BMUP1	BMUP2	BMUP3	BMUP4	*κ* (95%CI)
Residents	One	7	12	34	45	0.857 (0.793–0.957)
	Two	6	13	37	42	

Attending physicians	One	8	13	34	43	0.891 (0.815–0.968)
	Two	9	10	35	44	

Between group	Residents	7	12	36	43	0.845 (0.756–0.935)
	Attending physicians	9	12	35	42	

BMUP: bone marrow uptake pattern, BMUP1: direct invasion, BMUP2: focal/multifocal uptake, BMUP3: diffuse and homogeneous uptake, BMUP4: diffuse and heterogeneously uptake, and CI: confidence interval.

**Table 2:** Clinical characteristics of different^18^F-FDG PET/CT bone marrow uptake patterns.

	Total	BMUP1	BMUP2	BMUP3	BMUP4	*P*
Age	3.53 ± 2.17	2.97 ± 3.08	3.98 ± 2.57	2.42 ± 1.31	4.45 ± 2.02	＜0.001^*∗*^

*Gender*						
Female	50 (51.0%)	7 (77.8%)	5 (41.7%)	19 (54.3%)	19 (45.2%)	0.316
Male	48 (49.0%)	2 (22.2%)	7 (58.3%)	16 (45.7%)	23 (54.8%)	

*Primary site*						
Chest	13 (13.3%)	6 (66.7%)	1 (8.3%)	2 (5.7%)	4 (9.5%)	＜0.001^*∗*^
Abdomen	82 (83.6%)	3 (33.3%)	11 (91.7%)	30 (85.7%)	38 (90.5%)	
Pelvic	3 (3.1%)	0 (0.0%)	0 (0.0%)	3 (8.6%)	0 (0.0%)	

*INRGSS*						
L1	11 (11.2%)	0 (0.0%)	0 (0.0%)	11 (31.4%)	0 (0.0%)	＜0.001^*∗*^
L2	17 (17.3%)	5 (55.6%)	0 (0.0%)	12 (34.3%)	0 (0.0%)	
M	68 (69.4%)	4 (44.4%)	12 (100.0%)	10 (28.6%)	42 (100.0%)	
c	2 (2.1%)	0 (0.0%)	0 (0.0%)	2 (5.7%)	0 (0.0%)	

*INRGCS*						
Extremely low risk	12 (13.2%)	0 (0.0%)	0 (0.0%)	12 (37.5%)	0 (0.0%)	<0.001^*∗*^
Low risk	6 (6.6%)	4 (57.1%)	0 (0.0%)	2 (6.3%)	0 (0.0%)	
Medium risk	11 (12.1%)	2 (28.6%)	1 (9.1%)	7 (21.9%)	1 (2.4%)	
High risk	62 (68.1%)	1 (14.3%)	10 (90.9%)	11 (34.3%)	40 (97.6%)	

INRGSS: International Neuroblastoma Risk Group Staging System; INRGCS: International Neuroblastoma Risk Group Classification System; BMUP: bone marrow uptake pattern; BMUP1: direct invasion; BMUP2: focal/multifocal uptake; BMUP3: diffuse and homogeneous uptake; BMUP4: diffuse and heterogeneously uptake; ^*∗*^*P* < 0.05.

**Table 3:** BMI positive patients, BMI negative patients, PPV, NPV, sensitivity and specificity of four tests.

	BMI positive	BMI negative	PPV (%)	NPV (%)	Sensitivity (%)	Specificity (%)
PET/CT	70	28	92.9	92.9	97.0	83.9
BMB	41	57	100.0	54.4	61.2	100.0
PHOX2B of blood	48	50	95.8	58.0	68.7	93.5
PHOX2B of BM	62	36	96.7	80.6	89.6	93.5

BMI: bone marrow involvement, BMB: bone marrow biopsy, PHOX2B of blood: paired-like homeobox 2b of blood, PHOX2B of BM: paired-like homeobox 2b of bone marrow, PPV: positive predictive value, and NPV: negative predictive value.

**Table 4:** The diagnostic efficiency of different tests and compared with PET/CT.

	AUC	95%CI	*P*
PET/CT	0.904	0.828–0.955	—
BMB	0.806	0.714–0.879	0.028^*∗*^
PHOX2B of blood	0.811	0.719–0.883	0.078
PHOX2B of BM	0.916	0.842–0.962	0.817

BMB: bone marrow biopsy, PHOX2B of blood: paired-like homeobox 2b of blood, PHOX2B of BM: paired-like homeobox 2b of bone marrow, AUC: area under curve, and CI: confidence interval; ^*∗*^*P* < 0.05.

**Table 5:** The diagnostic accuracy of different tests based on the BMUP grouping and compared with PET/CT.

	BMUP1			BMUP2			BMUP3			BMUP4		
	Correct	Incorrect	*P*	Correct	Incorrect	*P*	Correct	Incorrect	*P*	Correct	Incorrect	*P*
PET/CT	6	3	—	12	0	—	31	4	—	42	0	—
BMB	3	6	0.347	2	10	＜0.001^*∗*^	28	7	0.324	39	3	0.240
PHOX2B of blood	3	6	0.347	4	8	0.001^*∗*^	29	6	0.495	39	3	0.240
PHOX2B of BM	7	2	＜1.000	10	2	0.478	30	5	＜1.000	42	0	1.000

BMB: bone marrow biopsy, PHOX2B of blood: paired-like homeobox 2b of blood, PHOX2B of BM: paired-like homeobox 2b of bone marrow, BMUP: bone marrow uptake pattern, BMUP1: direct invasion, BMUP2: focal/multifocal uptake, BMUP3: diffuse and homogeneous uptake, and BMUP4: diffuse and heterogeneously uptake; ^*∗*^*P* < 0.05.

## Data Availability

The data used in this study are available upon reasonable request from the corresponding author.

## Supplementary Materials

Supplementary Materials Supplementary Table 1: multiple comparisons of clinical characteristics. Supplementary Table 2: sensitivity, specificity, and accuracy of BMUP3. Supplementary Table 3: the sensitivity and specificity of different cut-off values in BMUP3. .
