# Supplementary material for: The Diagnostic Value of 18F-FDG PET/CT Bone Marrow Uptake Pattern in Detecting Bone Marrow Involvement in Pediatric Neuroblastoma Patients
Source: Contrast Media Mol Imaging. 2022 Jan 6;2022:7556315. doi: 10.1155/2022/7556315 (PMC8758298; doi:10.1155/2022/7556315)
Supplement: Supplementary Materials — Supplementary Table 1: multiple comparisons of clinical characteristics. Supplementary Table 2: sensitivity, specificity, and accuracy of BMUP3. Supplementary Table 3: the sensitivity and specificity of different cut-off values in BMUP3. . [file 7556315.f1.docx]

Supplementary Materials for

**The diagnostic value of 18F-FDG PET/CT bone marrow uptake pattern in detecting bone marrow involvement of pediatric neuroblastoma patients**

Jun Liu^1^, Cuicui Li^1^, Xu Yang^1^, Xia Lu^1^, Mingyu Zhang^1^, Luodan Qian^1^, Wei Wang^1^, Ying Kan^1^, Jigang Yang^1^*

Corresponding author: Jigang Yang^1^

E-mail: yangjigang@ccmu.edu.cn

Tel: 18811695983

^1^ Department of Nuclear Medicine, Beijing Friendship Hospital, Capital Medical University, Beijing 100050, China

Supplementary table 1: Multiple comparisons of clinical characteristics

|  |  | BMUP1 | BMUP2 | BMUP3 | BMUP4 |
| --- | --- | --- | --- | --- | --- |
| Age | BMUP1 | — | ＜1.000 | ＜1.000 | 0.058 |
|  | BMUP2 | — | — | 0.367 | ＜1.000 |
|  | BMUP3 | — | — | — | ＜0.001* |
|  | BMUP4 | — | — | — | — |
| Primary site | BMUP1 | — | 0.054 | 0.003* | 0.003* |
|  | BMUP2 | — | — | ＜1.000 | ＜1.000 |
|  | BMUP3 | — | — | — | 0.951 |
|  | BMUP4 | — | — | — | — |
| INRGSS | BMUP1 | — | 0.037* | ＜1.000 | ＜0.001* |
|  | BMUP2 | — | — | 0.001* | a |
|  | BMUP3 | — | — | — | ＜0.001* |
|  | BMUP4 | — | — | — | — |
| INRGCS | BMUP1 | — | 0.006* | 0.017* | ＜0.001* |
|  | BMUP2 | — | — | 0.048* | ＜1.000 |
|  | BMUP3 | — | — | — | ＜0.001* |
|  | BMUP4 | — | — | — |  |

INRGSS: International Neuroblastoma Risk Group Staging System, INRGCS: International Neuroblastoma Risk Group classification system, BMUP1: direct invasion, BMUP2: focal/multifocal uptake, BMUP3: diffuse and homogeneous uptake, BMUP4: diffuse and heterogeneously uptake, *P < 0.05, a: the same distribution is impossible for statistical comparison

Supplementary table 2: Sensitivity, specificity and accuracy of BMUP3

|  | Sensitivity | Specificity | Accuracy |
| --- | --- | --- | --- |
| BMUP3 | 71.4% | 92.6% | 88.6% |

BMUP3: diffuse and homogeneous uptake

Supplementary table 3: the sensitivity and specificity of different cut-off values in BMUP3.

|  | Cut-off value | Sensitivity | Specificity |
| --- | --- | --- | --- |
| BMUP3 | 1.00 | 100.0% | 0.0% |
|  | 1.50 | 100.0% | 33.3% |
|  | 2.08 | 71.4% | 92.6% |
|  | 3.00 | 14.3% | 100.0% |

BMUP3: diffuse and homogeneous uptake
